# Trace Elements in Food: Nutritional and Safety Issues

**DOI:** 10.3390/foods14162802

**Published:** 2025-08-13

**Authors:** Cristina Couto, Elisa Keating

**Affiliations:** 1 LAQV/REQUIMTE, Department of Chemical Sciences, Faculty of Pharmacy, University of Porto, Rua de Jorge Viterbo Ferreira 228, 4050-313 Porto, Portugal; 2 Associate Laboratory i4HB-Institute for Health and Bioeconomy, University Institute of Health Sciences-CESPU, 4585-116 Gandra, Portugal; 3 UCIBIO-Applied Molecular Biosciences Unit, Forensics and Biomedical Sciences Research Laboratory, University Institute of Health Sciences (1H-TOXRUN, IUCS-CESPU), 4585-116 Gandra, Portugal; 4 RISE-Health, Department of Biomedicine–Unit of Biochemistry, Faculty of Medicine, University of Porto, Alameda Prof. Hernâni Monteiro, 4200-319 Porto, Portugal; keating@med.up.pt

## 1. Introduction

In recent years, nutrition research has underscored the critical role of trace elements in food matrices. Although only required in microquantities, trace elements have profound physiological significance, including in the modulation of metabolism, immune responses, and supporting neurodevelopment. However, rising concerns regarding potential overexposure, contamination pathways, and limited bioavailability have led to intensified multidisciplinary investigations.

The nutritional relevance of trace elements extends beyond their intrinsic presence in raw food, encompassing alterations induced by various food processing techniques—including cooking, freezing, and leaching—which can influence concentration, stability, and safety. These considerations are central to public health and food quality assessment, and they warrant rigorous scrutiny in diverse dietary contexts. Motivated by these scientific and regulatory challenges, the current Special Issue of Foods, entitled “Trace Elements in Food: Nutritional and Safety Issues”, presents six original research articles addressing trace element characterization across a wide range of food matrices. The studies focus on octopus, eggs, rice, edible insects, and different types of coffee (espresso, ground, and instant), offering valuable insights into elemental profiles, metabolic implications, and the influence of culinary and industrial practices.

These contributions emphasize the complexity of trace element dynamics in current food systems and highlight the need for continued research to balance nutritional efficacy with toxicological safety.

## 2. An Overview of Published Papers

Ricardo Prego and co-workers (contribution 1) discussed the elemental concentration changes in edible (arm and mantle) and inedible (viscera) tissues of octopus (*Octopus vulgaris*) in raw and processed samples. The size of the octopus, process of cooking, and type of storage were considered and compared. The authors emphasized that the mechanism of variations in elemental concentrations subsequent to the cooking process and being stored frozen should be further studied.

Coffee was studied in contributions 2 and 6. A comparative trace element assessment of espresso coffee from Poland and Portugal analyzed samples collected from public cafés in both countries (contribution 2). The mineral profile of the espresso and the drinking water used to prepare it was determined and espresso samples from the two countries were found to be quite similar. For the majority of trace elements analyzed, espresso exhibited concentrations significantly higher than those found in the corresponding water. Notably, calcium (Ca) and strontium (Sr) constituted exceptions, as their presence in the final coffee beverage was primarily attributable to the mineral content of the water used in espresso preparation. Non-essential elements such as nickel (Ni) and lead (Pb) were detected in notable concentrations within the analyzed espresso samples. Previous research has indicated that these metals may leach from coffee brewing equipment, underscoring the need for further investigation to corroborate these findings.

Kamila Pokorska-Niewiada (contribution 6) assessed the quality of ground and instant coffee available on the Polish market, focusing on the concentration of six trace elements. The findings of this study demonstrate that a single cup of coffee poorly contributes to trace elements to dietary intake. Specifically, coffee prepared from 6.33 g of ground beans yields approximately 0.08–1.52% of the recommended daily allowance (RDA), whereas the same quantity of instant coffee contributes to 0.46–13.01% of the RDA, depending on the specific trace element evaluated. The minimal migration of lead (Pb) and cadmium (Cd) into the brewed coffee from the analyzed samples indicates that consumption, even up to six cups per day, poses no significant health risk to consumers. It is stressed that other types of coffee, and coffee additives that are becoming increasingly popular, should be further studied.

Contribution 3 aimed to differentiate eggs from backyard-raised hens compared to barn-raised eggs. Elemental profile and fatty acids profile analysis were described. This study demonstrated the efficacy of merging among isotopic, elemental, and fatty acid profiles, combined with chemometric techniques, in identifying backyard-raised hen eggs from eggs obtained from hens raised in barns. The backyard-rearing system was shown to offer distinct nutritional advantages, notably enhancing the quality of animal-derived food products.

Another study determined the total arsenic concentration (tAs) in rice and the most widely consumed rice-derived food products (white and brown rice, rice cakes, and rice noodles) available in Poland (contribution 4). Across all tested products, the inorganic arsenic (iAs) levels were below the maximum permissible limits, indicating no health risks for the adult population in Poland. However, a possible concern was identified for infants and young children. The authors recommend consumers to diversify their cereal intake and to opt for products exhibiting lower arsenic contamination.

The popularity of edible insects is on the rise, offering an environmentally friendly alternative and acting as protein-substitutes for traditional meat products. Yulianna Holowaty and co-workers (contribution 5) described the development and validation of an analytical method for the simultaneous assessment of a wide panel of trace elements (Al, As, B, Ba, Ca, Cd, Co, Cr, Cu, Fe, K, Mg, Mn, Mo, Na, Pb, Se, Sr, and Zn) in a variety of edible insects, scorpions, and tarantulas. The study determined that most of the samples were safe to consume for both children and adults at relatively high concentrations (1000 to 100,000 insects/day). However, the safe consumption limit for scorpions is considerably lower (around 30 individuals for adults and 10 for children). In contrast, tarantulas should be consumed in even more limited quantities, with a recommended intake of just one per day for children and two to three for adults. The sample’s heterogeneity was a point to be further investigated.

The papers published in this Special Issue emphasize the importance and necessity of additional investigation of trace elements in this field, with special attention to health safety and human biomonitoring. As guest editors, we sincerely hope this Special Issue serves as a valuable resource for advancing future research, and we extend our sincere appreciation to all contributing authors for their expert input.

## Data Availability

No new data were created or analyzed in this study. Data sharing is not applicable to this article.

